# METRICS: a pattern language of scholarship in medical education

**DOI:** 10.15694/mep.2017.000199

**Published:** 2017-11-07

**Authors:** Rachel Ellaway, David Topps

**Affiliations:** 1 University of Calgary

**Keywords:** scholarship, activity metrics

## Abstract

This article was migrated. The article was marked as recommended.

Scholarly activity in health professions education has been growing steadily but despite the broad interest, quite what is considered to be scholarly activity in medical education has remained vague. Boyer’s classes of scholarly activity ( Boyer 1990) and Glassick et al.’s criteria required of an artefact to render it scholarly ( Glassick et al. 1997) have been widely discussed. While the Glassick model has helped to define to what scholarly activity should be, we have found the Boyer model of what kinds of activity count as scholarship is lacking. We have developed the METRICS model of scholarly activity in medical education that maps more directly to scholarly activities.

- Metascholarship - activities that reflect on the nature of scholarship
- Evaluation - activities that measure value or axiology
- Translation - activities that move findings or practices from one domain to another
- Research - activities that focus on theory generation or testing (experimental, descriptive or explanatory)
- Innovation - activities that focus on creating new ideas, objects and practices
- Conceptual - activities that explore or develop new models, concepts, and paradigms
- Synthesis - activities that focus on the integration of existing knowledge and practice

Having built the METRICS model and tested it extensively in our own practice, we now seek to engage others in its use and appraisal.

## Introduction

The pursuit of scholarly activity in health professions education has been growing steadily since the 1990s, both in terms of the numbers and scope of the people involved in it, in terms of the quantity of work being generated, and in terms of the projected evidence-informed practice that this should enable. Despite the broad interest, quite what is considered to be scholarly activity in medical education has remained rather vague. More specifically, some activities, such as research, are less ambiguously seen as scholarly. Others, such as evaluation and innovation, are often not. This is not a new problem. Boyer’s classes of scholarly activity ( Boyer 1990) and Glassick et al.’s criteria required of an artefact to render it scholarly ( Glassick et al. 1997) have been widely discussed. However, while the Glassick model has helped to define to what scholarly activity should be, we have found, in teaching and mentoring others in educational scholarship, the Boyer model of what kinds of activity count as scholarship is somewhat lacking in articulating the breadth of activity in our field. Our response has been to develop a model of scholarly activity in medical education that maps more directly to the kinds of activities that are found in medical education. This paper sets out this model and describes some of its uses in advancing medical education scholarship.

## Background

The Glassick model ( Glassick et al. 1997), which set out the criteria within scholarship (clear goals, adequate preparation, appropriate methods, significant results, effective presentation, and reflective critique), has proved useful in articulating the values of scholarship but it does not focus on what can count as scholarly acts. Boyer’s model on the other hand, while it does focus on the ‘what’ of scholarship (discovery, integration, application, and teaching and learning), needs much translation to render it useful and, as such, is somewhat lacking in helping novice scholars to orient themselves to the field or in helping established scholars articulate what they have crafted (particularly when engaging others outside their field, such as in promotion applications). While these two models are the most often cited, there are others. For instance, Crites et al. (2014) proposed two distinct dimensions in medical education: ‘educational discovery (research) and teaching scholarship’ within the field. In summary, despite exploring a range of different models of scholarship, we found no single model that met our needs in articulating a practical pattern language of scholarly acts in medical education. This process did help, however, in clarifying our needs, central to which was a requirement for a simple and inclusive model of the different kinds of scholarship that legitimately make up the field of health professions education scholarship.

## Developing a model

**Context:** Our work in the Office for Health and Medical Education Scholarship at the University of Calgary involves mentoring and supporting both new and existing scholars in the field. We are also involved in graduate medical education scholarship training and in a range of editorial roles and activities that also need clarification regarding what kinds of scholarly acts are to be found in medical education.

**Figure 1.**
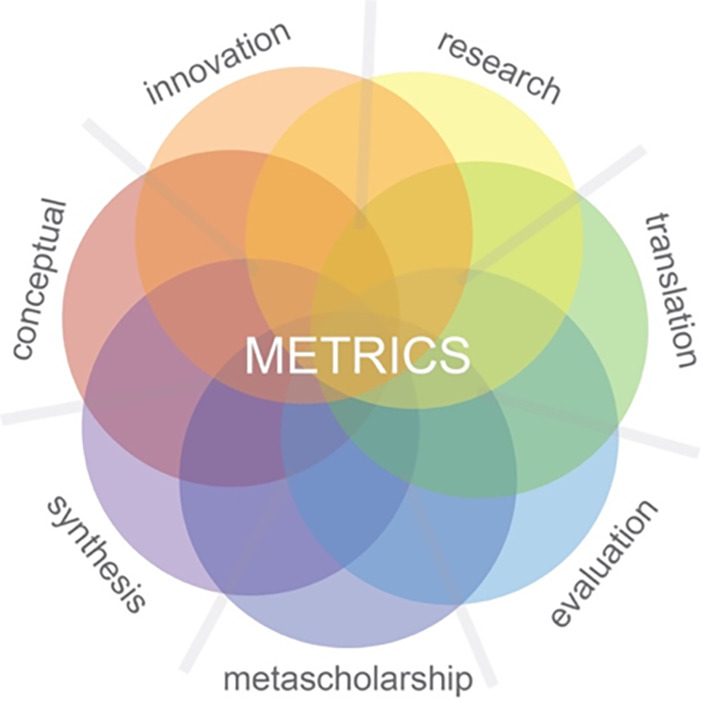
the METRICS model

**Process:** Our starting point was to consider the different varieties of scholarly acts that are commonly encountered in our field and from this to assemble a list of different scholarly approaches and activities. This list was iteratively grouped and simplified through discussion with members of our academic communities and through the use of early versions of the model in our teaching and professional development activities. This was guided by identifying common characteristics that included the purpose of the inquiry, its underlying assumptions and philosophy, its common procedural activities, and the discourses in the literature that conveyed both its legitimacy and how it differed from other kinds of scholarly acts. At the end of this process, we had a stable model of seven intersecting patterns of scholarly activity. We called this resulting model ‘METRICS’ - see Figure 1.

## The METRICS model

METRICS consists of seven intersecting patterns of scholarly activity:

• **Metascholarship:** Activities that explore or reflect on the nature of scholarly activities. These can include bibliographic inquiry (such as publication patterns or trends), reflections on the nature of scholarship in a field (such as proposing a research agenda), or on the development of scholarship and scholarly activities. For instance, this paper is an example of metascholarship. This pattern also includes work on scholarly leadership, professionalism of scholars, and scholarly career development.
• **Evaluation:** Activities that explore the value or axiology of things. Evaluation activities often focus on supporting decision-making and practice rather than advancing the field at a more abstract level and generate value statements that are either binary (good/bad, pass/fail etc.), or based on a comparison of different states, or a continuum between two extremes.
• **Translation:** Activities that translate practices or knowledge from one domain to another. These can include translating research findings into practice, translating concepts or perspectives from other disciplines to medical education, and translating concepts from one aspect of medical education to others, such as translating the OSCE model from assessment to admissions to create the MMI ( Eva et al. 2009).
• **Research:** Activities that focus on theory generation or testing. Research, in the context of this model, is framed as a more specific range of practices than is often the case in other circumstances when it is applied to mean any kind of systematic knowledge generation. Research can include any acts that are experimental, descriptive, or explanatory that involve a dialogical relationship between empiricism and theory.
• **Innovation:** Activities that focus on creating new ideas and things including the development of new instruments, techniques, practices, or organizations (of things, people, and ideas). Innovation scholarship may be summative (focused on the products and outputs of innovation) or formative (focused on the processes and experiences of innovation). Compared to other applied fields (such as engineering or computer science), the field of medical education has seen relatively little pure innovation scholarship published due to the expectation that any such study will, at the very least, include some aspect of evaluation of the innovation. Where this kind of work has been published (such as in the ‘what works’ or ‘really good stuff’ article formats) it has often been in short article formats rather than the full descriptive papers typically found in other fields.
• **Conceptual:** Activities that explore or develop the conceptual basis of a field. Examples include thought experiments, the deductive development of new models, concepts, and paradigms, critiques of exiting practices, thought and opinion papers, and reflection papers (such as Medical Education’s ‘when I say’ format). Other examples would include calls for educational reform, and papers exploring tensions and problems in medical education.
• **Synthesis:** Activities that focus on existing knowledge and practice. These include any secondary activity (that synthesizes primary scholarly materials) or tertiary activity (that synthesizes other syntheses) as well as material on the nature of knowledge synthesis in general. Examples can include; literature reviews, scoping reviews, systematic reviews, and meta-analyses, as well as techniques for conducting effective acts of synthesis.

In considering the METRICS model, it should be understood that these seven patterns are not a taxonomy where a scholar has to pick one to the exclusion of the rest as their frame of reference for a particular act of scholarship (research protocol, conference presentation, journal paper etc.). A typical research paper will include a literature review (synthesis), some form of instrument or intervention development (innovation), followed by data capture, analysis and theory development (research). Similarly, scoping and systematic reviews typically involve elements of synthesis, metascholarship, and evaluation. We can therefore understand METRICS as a pattern language for acts of educational scholarship rather than a typology of domains of scholarly activity ( Alexander et al. 1977). It is a hallmark of pattern thinking that an instance of a pattern does not need to reflect all of the prototypical pattern’s characteristics; it only needs to exhibit enough of them to be recognizable as a member of the pattern. To that end, a relatively discrete set of pattern concepts can be used to model a wide range of specific examples. It is the expression of each pattern and the combinations and interactions of these patterns that makes each instance unique. Patterns are an epistemological approach to describing complex and ill-defined systems, such as medical education scholarship ( Ellaway and Bates 2015).

## Using the METRICS model

The METRICS pattern language can be applied in many settings and activities. For instance, we have used it in our teaching both about scholarship and how to engage in scholarly activity in general, as well as in instruction around particular approaches to scholarship in medical education. The model has also proved useful as part of our mentorship and leadership activities in the field. Of particular note is the utility of the model in helping developing scholars to understand the different kinds of scholarly activity required within their studies, projects, and programs of inquiry. It has also been useful in critical appraisal of the medical education literature and in structuring peer reviews of papers and grant proposals.

METRICS has parallels with competency models, such as CanMEDS, in that each of the METRICS patterns is a role, an approach to practice, that medical education scholars typically engage in. Indeed, one of the more critical implications of METRICS is that being a scholar in our field means being able to function credibly in the seven areas, even if some are pursued more or less than others. METRICS can therefore be a way of articulating professionalism of practice in medical education scholarship in terms of what scholars should be able to do, and not just the values they are expected to follow in plying their trade.

The question of how useful this model is to others has yet to be explored. Our publishing this model is the first step in advancing this process. Although we have used METRICS extensively within our own institution and across a range of our own activities, we do not claim global applicability and seek instead to engage in conversations within the community around the issues that the model may raise. For instance, further validation of a model should explore to what extent the model is stable across different contexts (it is not broken by new examples) and it is useful (the model helps learners and practitioners with their work). It would also be an indicator of the model’s utility.

## Next steps

Having built the METRICS model and tested it extensively in our own practice, we now seek to engage others in its use and appraisal. How others will do this and the extent to which this happens is outside the scope of this paper. However, we believe that the METRICS pattern language has great potential to support the development and pursuit of scholarship in medical education. To that end, we propose the model and our approach in developing it to the medical education community.

## Take Home Messages

• Current models describing scholarly activities in medical education are lacking.
• We find that our METRICS model and pattern language is more helpful in describing such activities.
• We encourage discourse in the medical education community on the metrics of the METRICS model.

## Notes On Contributors

Rachel Ellaway is Professor in medical education in the Department of Community Health Sciences and the Director of the Office of Health and Medical Education Scholarship (OHMES), Cumming School of Medicine, University of Calgary, Alberta, Canada.

David Topps is Professor in the Department of Family Medicine and the Medical Director of the Office of Health and Medical Education Scholarship (OHMES), Cumming School of Medicine, University of Calgary, Alberta, Canada.
