# Supplementary material for: METRICS: a pattern language of scholarship in medical education
Source: MedEdPublish (2016). 2017 Nov 7;6:199. [Version 1] doi: 10.15694/mep.2017.000199 (PMC10885223; doi:10.15694/mep.2017.000199)
Supplement: Peer review history

# Reviewer report: Reviewer response for version 1

**DOI:** 10.21956/mep.19387.r28413

**Published:** 2017-11-16

**Authors:** Susan Van Schalkwyk

**Affiliations:** 1 Stellenbosch University

This review has been migrated. The reviewer awarded 5 stars out of 5 I was immediately attracted to the title of this article when it arrived in my inbox and it did not disappoint. Building on the seminal work of Boyer and later Glassick, the model that these authors have put forward makes sense in many ways and I can see it serving as a most useful tool in a number of different contexts (research methodology courses, writing retreats, postgraduate supervision and the like. I particularly liked the richer understanding of 'evaluation' and its potential to advance the field. Many readers of MedEdPublish will also intuitively response to the idea of 'pattern thinking' and that one seeks patterns across a set of characteristics, rather than adopting an approach of categorising discrete activities. This provides a more encompassing idea of scholarship, while at the same time offering the reader clear definitions to work with in a practical way.

Reviewer Expertise:

NA

No decision status is available

# Reviewer report: Reviewer response for version 1

**DOI:** 10.21956/mep.19387.r28417

**Published:** 2017-11-07

**Authors:** Subha Ramani

**Affiliations:** 1 Harvard Medical School, Brigham and Women's Hospital

This review has been migrated. The reviewer awarded 5 stars out of 5 This paper on scholarship is a must read for every Health Professions Educator (HPE) Scholar and those who mentor scholars. It is written by experts in the field who have thoughtfully analyzed existing models, the gaps within those models, and articulated the need for a model that can be understood and used by HPE scholars. with growing HPE training programs including Masters and Doctorate degree programs, this model will be useful in guiding scholarly activities and products of trainees. I hope educational leaders around the world apply this model in their own setting and document its value and challenges.I have only one comment. Some sentences in the introduction section are complex, with multiple clauses, I saw one instance of a double negative (are less ambiguously seen). It may be challenging for readers, whose primary language is not English, to process and grasp quickly.All in all, an outstanding paper with a new and possibly more easily applicable scholarship model. I strongly recommend this paper and will be adding it to my syllabus on scholarship.

Reviewer Expertise:

NA

No decision status is available

# Reviewer report: Reviewer response for version 1

**DOI:** 10.21956/mep.19387.r28418

**Published:** 2017-11-07

**Authors:** Trevor Gibbs

**Affiliations:** 1 AMEE

This review has been migrated. The reviewer awarded 5 stars out of 5 There are some acronyms that are novel, some even funny but many that lack practicality, this is not one of them.The METRICS model is a very practical approach to scholarship structure and I really appreciate the circular spectrum of activities rather than a linear progression through individual markers of scholarship. As the authors point out, the proof of the pudding is in the eating and evaluating how this model can work in different contextsI strongly recommend this paper to those involved at all levels within academic structures, many of which still struggle with the concept of scholarship and look forward to reading subsequent papers that demonstrate and evaluate its use

Reviewer Expertise:

NA

No decision status is available

# Reviewer report: Reviewer response for version 1

**DOI:** 10.21956/mep.19387.r28414

**Published:** 2017-11-07

**Authors:** Ronald M Harden

**Affiliations:** 1 AMEE

This review has been migrated. The reviewer awarded 5 stars out of 5 In a book to be published next year we suggest that all teachers have a role as a scholar. Existing definitions and descriptions such as the Boyer definition are not helpful. It is great to see this practical framework which should be read by all teachers.

Reviewer Expertise:

NA

No decision status is available

# Reviewer report: Reviewer response for version 1

**DOI:** 10.21956/mep.19387.r28416

**Published:** 2017-11-07

**Authors:** Richard Hays

**Affiliations:** 1 James Cook University

This review has been migrated. The reviewer awarded 5 stars out of 5 This is an excellent summary of the current view on scholarship in medical education and it should be read by all budding medical educators. I can think of only one possible additional comment. At risk of upsetting pedantic spellers, I would like to see another 'T' in there. This is for theory-building, because something that scholarship must do is contribute to new theory development as well as application of existing theory. I have hard rather cruel statement such as that medical education is 'a set of practices chosen on personal whim without a theoretical base'. While I do not agree with that, we have a responsibility to define, document, evaluate and disseminate when we practice/ develop/ innovate/ apply and learn from experience. The authors do kind of include this under conceptual development, but perhaps it could be a little more prominent?

Reviewer Expertise:

NA

No decision status is available

# Reviewer report: Reviewer response for version 1

**DOI:** 10.21956/mep.19387.r28415

**Published:** 2017-11-07

**Authors:** P Ravi Shankar

**Affiliations:** 1 American International Medical University

This review has been migrated. The reviewer awarded 5 stars out of 5 I enjoyed reading this thought provoking paper about medical education research and scholarship. The authors have put forward a model which can address various research activities in the field of medical and health professions education research. Medical education often deals with abstract entities and concepts with important real life implications. The authors have described the METRICS model well using examples and various categories of papers from the literature. I agree with the authors that these components may often overlap and a research paper can have multiple components. This paper will be very useful for medical educators, individuals pursuing higher degrees and fellowships in medical education and those actively involved in research in this field.

Reviewer Expertise:

NA

No decision status is available
